# Evidence synthesis of effective e-cigarette prevention messages for adolescents and young adults: A scoping review

**DOI:** 10.18332/tid/208097

**Published:** 2025-07-30

**Authors:** Yu Chen, Haoyi Liu, Shiyu Liu, Jing Xu, Xinyao Yu, Yujiang Cai, Si Chen, Ying Wang

**Affiliations:** 1 School of Art and Communication, Fujian Polytechnic Normal University, Fuqing, China; 2 College of Liberal Arts and Social Sciences, City University of Hong Kong, Hong Kong SAR, China; 3 School of Public Health, Xi’an Jiaotong University, Xi’an, China; 4 School of Journalism and Communication, Peking University, Beijing, China; 5 Faculty of Humanities and Arts, Macau University of Science and Technology, Macau, China; 6 School of International Studies, Peking University, Beijing, China; 7 China National Center for Food Safety Risk Assessment, Beijing, China; 8 School of Humanities and Communication, Zhejiang University of Finance and Economics, Hangzhou City, China

**Keywords:** adolescents, e-cigarettes, prevention, information design, scoping review

## Abstract

**INTRODUCTION:**

This scoping review aims to examine existing evidence regarding information design for youth e-cigarette prevention, identify research gaps, and provide recommendations for future research and practice.

**METHODS:**

A comprehensive literature search was conducted from the inception of databases to April 2024 across six databases: Web of Science Core Collection (including MEDLINE), PubMed, Embase, Scopus, PsycINFO, and CNKI. Peer-reviewed articles related to information design for youth e-cigarette prevention were included based on eligibility criteria. Two reviewers independently screened articles, extracted data, and synthesized results following PRISMA-ScR guidelines.

**RESULTS:**

Thirty studies met the inclusion criteria. Most studies were conducted in the United States (n=28) and employed quantitative methods (n=20). Gain-loss framing was the most commonly used theoretical framework. Three core themes in youth e-cigarette prevention information design were identified: emphasizing usage risks, optimizing presentation methods, and segmenting target audiences. Primary outcome measures included perceived message effectiveness (PME) and e-cigarette-related knowledge, beliefs, attitudes, and behavioral intentions.

**CONCLUSIONS:**

Preliminary evidence provides guidance for youth e-cigarette prevention information design. Future research should evaluate message effectiveness across diverse populations, explore message customization strategies, assess behavioral outcomes, and strengthen theoretical foundations and applications.

## INTRODUCTION

Electronic cigarette use among adolescents has emerged as a significant global public health concern. Despite being marketed as a safer alternative to conventional cigarettes, e-cigarettes pose various health risks, particularly for young users whose brains are still developing^1^. Adolescent e-cigarette use has been associated with nicotine addiction, respiratory symptoms, and increased risk of initiating conventional smoking^2^. In the United States, e-cigarette use among middle and high school students rose dramatically between 2017 and 2019, with 27.5% of high school students and 10.5% of middle school students reporting e-cigarette use in the past 30 days^3^.The situation in China is equally concerning. A nationwide survey revealed that in 2021, 8.7% of students aged 14–17 years used e-cigarettes, with usage rates reaching 12.5% among high school students^4,5^. Another study found that e-cigarette use among Chinese university students increased from 0.9% in 2015 to 2.7% in 2019^6^.

The United Nations defines ‘youth’ as individuals aged 15–24 years and ‘children’ as those aged <14 years^7^. However, in the context of smoking prevention, considering a broader age range is crucial, as e-cigarette use often begins in early adolescence. Therefore, this study focuses on adolescents aged 10–24 years, encompassing both children and youth as defined by the United Nations^8^.

Given the rising prevalence and potential harms of adolescent e-cigarette use, effective interventions are needed to prevent its initiation and progression. Communication interventions are vital in curbing the e-cigarette epidemic among adolescents. Health communication campaigns are considered an effective approach to educating adolescents about e-cigarette risks and influencing their attitudes and behaviors^8^. Such campaigns aim to disseminate persuasive messages through various channels, including mass media, social media, and interpersonal communication, to reach and engage target audiences^9^. While mass media campaigns have proven successful in preventing conventional cigarette smoking among adolescents^9-11^, evidence for e-cigarette prevention remains limited^12^.

Several studies have explored information design strategies for adolescent e-cigarette prevention, yielding important insights. Qualitative research by Roditis et al.^13^ found that adolescents prefer messages emphasizing e-cigarette health risks, using vivid imagery, and featuring peer testimonials. Similarly, another survey identified messages focusing on addiction and health consequences as most effective in deterring adolescent e-cigarette use^14^. Experimental research by Noar et al.^15^ demonstrated that fear-arousing messages had a greater impact on adolescents’ risk perceptions and e-cigarette use intentions compared to neutral messages.

Despite growing research in this field, evidence regarding effective information design for adolescent e-cigarette prevention remains fragmented and uncertain, lacking comprehensive synthesis. Furthermore, the theoretical foundations of effective information design in this domain have not been systematically reviewed, limiting our understanding of the mechanisms by which messages influence adolescents’ e-cigarette-related attitudes and behaviors. Health communication theories, such as the Extended Parallel Process Model, the Elaboration Likelihood Model, and the Theory of Planned Behavior^16,17^, provide valuable frameworks for understanding how messages influence health-related attitudes, intentions, and behaviors. A review examining the application of these theories in adolescent e-cigarette prevention messaging can advance the development of theory-driven information design strategies^18^.

This study will conduct a scoping review to synthesize existing evidence on effective information design for adolescent e-cigarette prevention campaigns. Through comprehensive, critical assessment of the current research landscape, we aim to identify promising approaches and research gaps to inform evidence-based interventions. By integrating findings from diverse study designs and contexts, scoping reviews can provide more robust and generalizable conclusions than individual studies^19^. Using a scoping review methodology, this study addresses the following questions:

1. What are the general characteristics of existing research (e.g. publication years, sample populations, research methods, theoretical foundations, outcome measures)?
2. What prevention message content (themes, strategies) has been examined? What are the key findings?
3. What are the limitations and gaps in existing research? What are the implications for related research and practice?

## METHODS

This study followed the PRISMA-ScR guidelines and the Arksey and O’Malley^20^ five-step framework for literature search, screening, extraction, and synthesis^21^. The protocol was registered on the Open Science Framework (OSF) (https://doi.org/10.17605/OSF.IO/QK2B4).

### Inclusion and exclusion criteria

Studies meeting the following criteria were included: (Population) Children and youth aged 10–24 years, as defined by the United Nations; (Intervention) E-cigarette prevention messages designed for health communication campaigns; (Language) Published in English or Chinese; (Publication type) Peer-reviewed original research articles; (Publication time) Published studies from database inception to April 2024 were included without date restrictions to capture the full scope of available evidence.

Studies were excluded if they met any of the following criteria: not focused on e-cigarette prevention campaign design; adults aged ≥24 years; not related to e-cigarette prevention; and non-peer-reviewed research articles (e.g. reviews, commentaries, theses, conference papers). No geographical limitations were applied to ensure comprehensive global coverage.

### Search strategy

We conducted searches in Web of Science Core Collection (including MEDLINE), PubMed, Embase, Scopus, PsycINFO, and CNKI databases without language or time restrictions. The searches were performed in April 2024.

Search terms included variations of ‘electronic cigarette’, ‘youth’, ‘prevention’, ‘communication’, and ‘message’. Detailed search strategies for each database are provided in Supplementary file Material 1.

### Data extraction

**Figure 1:**
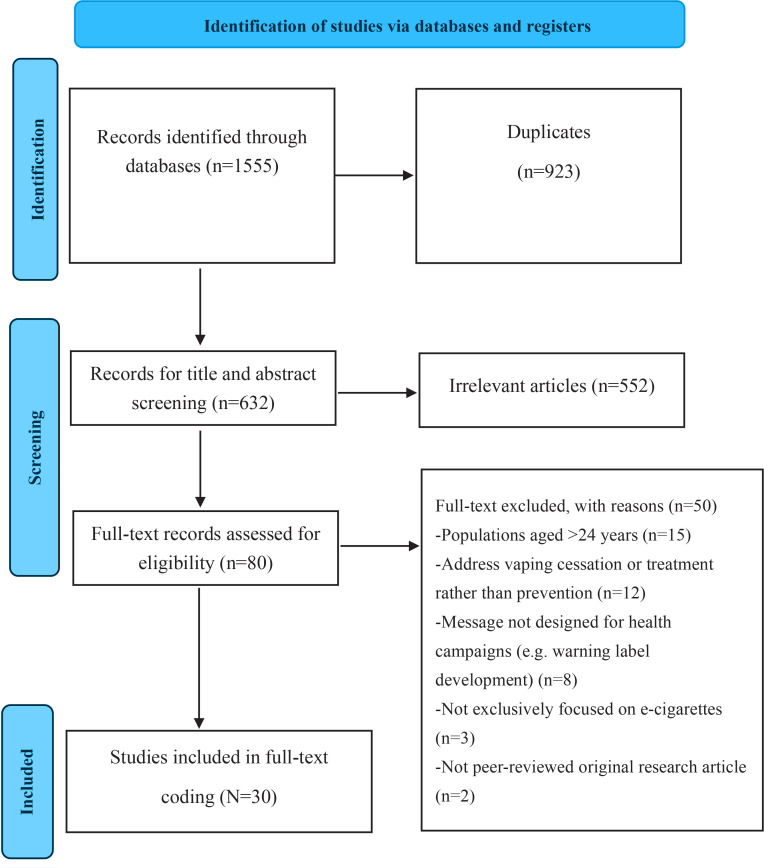
PRISMA flow diagram

Two independent researchers screened titles and abstracts, reviewed potentially relevant full texts, and extracted data from included studies. Any discrepancies were resolved through discussion or consultation with a third reviewer to minimize risk of bias. The screening process was summarized using a PRISMA flow diagram (Figure 1)^22^. Extracted data included article title, authors, country, research methodology, theoretical framework, study design, demographic characteristics, sample size, study materials, outcome measures, research objectives, findings, and study limitations.

### Data synthesis

Given the heterogeneity in study designs, message characteristics, and outcome measures, a descriptive qualitative synthesis was employed to analyze the extracted data. The analysis focused on identifying message themes and strategies that demonstrated potential effectiveness across multiple studies, examining theoretical foundations, methodological limitations, and implications for future research. Key characteristics are visually presented through figures and tables.

## RESULTS

### Study characteristics

Following screening of 1555 records and removal of duplicates, 632 titles and abstracts were reviewed. After full-text assessment of 80 articles, 30 studies met the inclusion criteria (Figure 1).

As shown in Table 1, the majority of studies were conducted in the United States (n=28), with single studies from Australia (n=1) and Canada (n=1). The 30 articles were published between 2016 and 2023, primarily in health communication and public health journals, including *Addictive Behaviors* (n=7), *Health Communication* (n=3), and *Health Promotion Practice* (n=2).

**Table 1:** Study characteristics of the evidence synthesis of effective e-cigarette prevention messages for adolescents and young adults (N=30)

*Category*	*n (%)*
**Publication year**	
2016	1 (3.3)
2019	3 (10)
2020	5 (16.7)
2021	8 (26.7)
2022	3 (10)
2023	9 (30)
2024	1 (3.3)
**Tobacco and e-cigarette use**	
E-cigarette users	25 (83.3)
Never used e-cigarettes	6 (20)
Smokers	11 (36.7)
**Research method**	
Quantitative	20 (66.7)
Qualitative	8 (26.7)
Mixed methods	2 (6.7)
**Theory**	
Framing theory (gains/losses)	3 (10.0)
Narrative persuasion theory	2 (6.7)
Prospect theory	2 (6.7)
Other theories	7 (23.3)
Not reported	17 (56.7)
**Race/ethnicity**	
White	24 (80)
Black	18 (60)
Hispanic/Latino	18 (60)
Asian	13 (43)
Other	13 (4)
**Country**	
USA	28 (93.3)
Australia	1 (3.3)
Canada	1 (3.3)
**Age** (years)	
10–18	12 (40)
18–24	9 (30)
Both age groups	9 (30)
**Information type**	
Text only	14 (46.7)
Image only	1 (3.3)
Video/advertisement	8 (26.7)
Image and text (e.g. posters, print ads)	8 (26.7)
Other formats	5 (16.7)

Methodologically, quantitative approaches predominated, with 20 quantitative studies, 8 qualitative studies, and 2 mixed-methods studies. Research designs included cross-sectional surveys, online experiments, and focus groups. Sample sizes ranged from 27 to 6427 participants.

Regarding theoretical frameworks, the majority of studies (n=17; 56.7%) did not specify a theoretical foundation. Among the 13 studies (43.3%) that referenced theoretical frameworks, gain-loss framing theory (n=3), prospect theory (n=3), and narrative persuasion theory (n=2) were most frequently cited. Notably, while some studies mentioned theoretical frameworks, they often failed to systematically apply these theories to guide research design or test theoretical hypotheses.

Demographic characteristics revealed that 12 studies focused on adolescents (aged 10–18 years), 9 on young adults (aged 18–24 years), and 9 included both age groups, primarily targeting college and secondary school students. Gender distribution was reported in 28 studies (93%), with female representation ranging from 40.2% to 75.4%, indicating relatively balanced gender ratios. While most studies included White participants (n=24), representation of Black, Asian, and Hispanic populations was comparatively low. Electronic cigarette use status was reported in 17 studies (n=25 for e-cigarette use, n=11 for tobacco use), with only 6 studies specifically recruiting never users of e-cigarettes. Additionally, 4 studies recruited urban samples, 2 included suburban populations, 2 included rural populations, while the remaining studies did not specify geographical context.

### Research materials and sample size

Among the 30 studies analyzed (Table 1), text-only prevention messages were most prevalent (n=14), followed by combined text-image materials (n=8) and video content (n=8), with standalone images being least common (n=1). The number of message stimuli varied considerably across studies, ranging from a few messages to 960 distinct items. Video durations typically spanned from several seconds to multiple minutes. For combined text-image materials, posters and print advertisements were predominant formats. Video content encompassed both television public service announcements and short-form digital videos.

### Outcome measures and effects

Perceived message effectiveness (PME) and perceived effectiveness (PE) emerged as the primary outcome measures, typically assessed through validated scales^15,23^. Additional frequently evaluated outcomes included: enhanced knowledge and awareness of e-cigarette risks and harms, strengthened beliefs about negative consequences, development of more negative attitudes toward e-cigarettes, and reduced intentions and susceptibility to e-cigarette use^24^.

### Electronic cigarette prevention message design themes

Through comprehensive analysis of 30 studies, three core themes emerged in electronic cigarette prevention message design: emphasizing usage risks and harms, optimizing presentation strategies and discourse approaches, and tailoring messages for specific populations. These themes provide guidance for content development, presentation format, and audience segmentation in prevention messaging.

The first theme focuses on message content design. Substantial evidence indicates that emphasizing the negative impacts of electronic cigarettes on adolescent physical and mental health is the most effective messaging approach^15,24,25^. These impacts include addiction risk, respiratory diseases, cardiovascular damage, and impaired brain development. Vivid presentations of these health hazards significantly enhance adolescents’ risk perception and reduce their intention to use electronic cigarettes. For example, Hoffman et al.^26^ found that exposing adolescents to television drama segments depicting electronic cigarette-induced lung injury strengthened their cognition and attitudes regarding electronic cigarette dangers. Beyond health risks, some studies revealed that highlighting social risks, such as damage to social image and interpersonal relationships, can be persuasive for certain adolescents^26^. In contrast, merely disclosing inappropriate marketing practices by the electronic cigarette industry, such as concealing ingredients or targeting youth, proved ineffective in deterring adolescents from electronic cigarette use^13,27^. These findings suggest that prevention message content should prioritize direct personal consequences rather than emphasizing external factors. In addition, fear-based messages demonstrated stronger immediate risk perception effects compared to narrative-based approaches, particularly among non-users^28^. However, narrative formats showed superior engagement and reduced psychological reactance among current users^29-31^. Demographic subgroup analysis revealed differential effectiveness: males responded more favorably to social risk messages emphasizing peer disapproval, while females showed greater receptivity to health-focused content detailing physical harms.

The second theme addresses effective delivery methods for prevention content to adolescent audiences. Traditional didactic or threatening approaches often fail to resonate with youth. Research indicates that narrative presentation formats, which employ engaging storylines and character development to evoke empathy and identification, facilitate adolescents’ reception and processing of prevention messages^28,32^. For instance, the Liu and Yang^31^ experiment found that embedding electronic cigarette hazard information within campus news stories enhanced risk perception and behavioral intentions through readers’ emotional connection with characters. Additionally, visual elements such as images, videos, and warning graphics can effectively illustrate health hazards and capture adolescent attention^9,28^. A content analysis of electronic cigarette-related YouTube videos revealed that content focusing on harm demonstration garnered more adolescent engagement^26^. Interactive elements also proved crucial. Incorporating question-and-answer sessions and mini-games can increase adolescent engagement and information retention. The Lazard^28^ social media interactive messages significantly improved adolescents’ knowledge and beliefs about electronic cigarette hazards. Notably, discourse strategies should be tailored to different populations^28^. For current electronic cigarette users, emphasizing cessation benefits (gain-framed) proves more effective, while for non-users, emphasizing usage risks (loss-framed) shows greater impact^25,33^. Narrative, visual, and interactive presentation formats consistently outperform traditional didactic approaches in engaging adolescent audiences.

The third theme emphasizes tailoring prevention messages to target audience characteristics. First, messaging style and presentation format should vary across age groups. Research demonstrates that younger adolescents prefer cartoon formats, while older adolescents respond better to celebrity endorsements^25,28^. Second, prevention emphasis should differ based on electronic cigarette usage status. For current users, messages should guide cessation processes and emphasize feasibility and benefits; for non-users, messages should focus on resistance strategies and abstinence^34,35^. Third, gender differences influence message reception. Studies reveal that male adolescents are more concerned about social recognition impacts, while females focus more on physical health hazards^33^. Consequently, messages targeting males should emphasize social risks, such as being labeled as ‘troubled youth’, while those targeting females should detail component hazards, including nicotine’s effects on fertility.

## DISCUSSION

This scoping review systematically searched, screened, and synthesized evidence regarding youth e-cigarette prevention message design in the United States, aiming to summarize the current research landscape, identify effective message characteristics including content and presentation strategies, evaluate assessment methods and theoretical foundations, and analyze research gaps to provide directional recommendations for future studies.

While we found increasing research attention to this topic, studies were conducted mainly in the USA (n=28), with single studies from Australia and Canada. The overwhelming US focus significantly limits generalizability to other sociocultural contexts with different regulatory frameworks, tobacco cultures, and risk perceptions. Cross-cultural research is urgently needed to understand how cultural values, social norms, and regulatory environments influence message effectiveness. International perspectives, such as the Ludovichetti et al.^36^ comparative study on youth risk perceptions across different nicotine products, highlight the need for culturally tailored messaging strategies that consider diverse societal contexts.

The methodological approaches primarily consist of quantitative online cross-sectional surveys and experiments. A critical limitation is the poor theoretical anchoring across studies. While 13 studies (43.3%) mentioned theoretical frameworks, few demonstrated rigorous theory-driven message design or empirically tested theoretical hypotheses. This theoretical deficit limits our understanding of the mechanisms by which messages influence adolescent attitudes and behaviors, and impedes the development of evidence-based intervention strategies. Our study revealed that 7 studies used PME as a primary outcome measure. While PME provides insights into message reception, it measures audience perceptions rather than actual behavior change or real-world prevention effectiveness. This creates uncertainty about whether promising message strategies translate to reduced e-cigarette initiation or use in practice. The disconnection between perceived effectiveness and behavioral impact represents a significant gap between research evidence and public health needs.

Our analysis identified three core elements in e-cigarette prevention message design: emphasizing usage risks, optimizing presentation methods, and segmenting target audiences. These themes are interconnected and collectively guide prevention message design and dissemination. Risk emphasis forms the content foundation, revealing e-cigarettes’ impacts on youth physical health, mental well-being, and social adaptation as essential prevention message components. Presentation methods serve as communication bridges, employing narrative, visual, and interactive formats that appeal to youth to enhance message attractiveness and persuasiveness. Audience segmentation provides implementation guidance, tailoring message content and style to different populations to improve targeting and effectiveness. These findings offer strategic guidance for future prevention efforts, suggesting that youth e-cigarette prevention campaigns should expand prevention message dimensions, innovate presentation formats, deepen audience segmentation, and maintain broad, sustained information dissemination to curb e-cigarette prevalence among youth.

### Limitations

This review has several limitations. Despite employing comprehensive search strategies, some uncatalogued literature may have been missed. The representativeness of included studies is limited, with samples predominantly restricted to US youth, lacking international comparative perspectives and affecting result generalizability. Research designs were primarily short-term and cross-sectional. The included studies showed substantial heterogeneity in population characteristics, message types, and measurement indicators, precluding quantitative synthesis. Some studies lacked complete transparency in reporting key information about design, implementation, and analysis, potentially affecting bias risk assessment. Following standard scoping review methodology, we focused on descriptively mapping the research landscape rather than systematically evaluating evidence quality.

## CONCLUSIONS

Future research should strengthen theoretical applications in prevention message design. Given the complexity of e-cigarette use intentions and behaviors, single theories may inadequately guide message design. Researchers should actively explore integrating multiple theoretical perspectives, combining theories from communication, psychology, and public health to comprehensively consider cognitive, emotional, and environmental factors. This integration would enhance theoretical precision in guiding message content and format planning while empirically testing theories to refine the theoretical framework for youth e-cigarette prevention campaigns. Methodologically, research paradigms should be innovated to improve theoretical alignment and methodological rigor, employing novel approaches such as longitudinal tracking designs and big data analytics to examine prevention messages’ behavior change mechanisms and actual effectiveness from multiple angles. Communication design strategies must evolve with youth media consumption habits, enhance interactive engagement, and center on youth audiences while respecting their agency and meeting differentiated needs. Research scope should be broadened to strengthen youth e-cigarette use and prevention studies across different cultural, social, and policy contexts, conducting international comparisons to explore commonalities and differences. Establishing multidisciplinary collaborations would translate research evidence into actionable message design principles, assessment standards, and practical guidelines, providing evidence-based references for youth e-cigarette prevention education and fostering positive research-practice interactions.

## Supplementary Material



## Data Availability

The data supporting this research are available from the authors on reasonable request.
