# Supplementary material for: Evidence synthesis of effective e-cigarette prevention messages for adolescents and young adults: A scoping review
Source: Tob Induc Dis. 2025 Jul 30;23:10.18332/tid/208097. doi: 10.18332/tid/208097 (PMC12309842; doi:10.18332/tid/208097)
Supplement: Supplementary file 1 [file TID-23-111-s1.pdf]

Material 1: Search Strategy

**1). PubMed**	
#	Searches
1	"Electronic Nicotine Delivery Systems"[MeSH Terms] OR ("electronic cigarette"[Title/Abstract] OR "e-cigarette"[Title/Abstract] OR "vape"[Title/Abstract] OR "vaping"[Title/Abstract] OR "electronic nicotine delivery system"[Title/Abstract]
2	"Child"[MeSH Terms] OR "Adolescent"[MeSH Terms] OR "Young Adult"[MeSH Terms] OR ("minor"[Title/Abstract] OR "underage"[Title/Abstract] OR "youth"[Title/Abstract] OR "Adolescent"[Title/Abstract] OR "teenager"[Title/Abstract] OR "Child"[Title/Abstract] OR "student"[Title/Abstract] OR "undergraduate"[Title/Abstract] OR "postgraduate"[Title/Abstract] OR "young"[Title/Abstract] OR "youngster"[Title/Abstract]
3	"Communication"[MeSH Terms] OR "Health Communication"[MeSH Terms] OR ("prevent"[Title/Abstract] OR "prevention"[Title/Abstract] OR "intervention"[Title/Abstract] OR "campaign"[Title/Abstract]) OR "Communication"[Title/Abstract]
4	"message"[Title/Abstract] OR "information"[Title/Abstract]
5	1 AND 2 AND 3 AND 4
**2). Embase**	
#	Searches
1	'electronic cigarette'/exp OR 'electronic cigarette':ti，ab，kw OR 'e cigarette':ti，ab，kw OR vape:ti，ab，kw OR vaping:ti，ab，kw OR 'electronic nicotine delivery system':ti，ab，kw
2	'adolescent'/exp OR 'child'/exp OR 'young adult'/exp OR adolescent:ti，ab，kw OR child:ti，ab，kw OR minor:ti，ab，kw OR

	underage:ti，ab，kw OR youth:ti，ab，kw OR teenager:ti，ab，kw OR student:ti，ab，kw OR undergraduate:ti，ab，kw OR postgraduate:ti，ab，kw OR young:ti，ab，kw OR youngster:ti，ab，kw
3	prevent:ti，ab，kw OR prevention:ti，ab，kw OR intervention:ti，ab，kw OR communication:ti，ab，kw OR campaign:ti，ab，kw
4	message:ti，ab，kw OR information:ti，ab，kw
5	1 AND 2 AND 3 AND 4

3). SCOPUS

#	Searches
1	TITLE-ABS-KEY(electronic cigarette OR e-cigarette OR vape OR vaping OR electronic nicotine delivery system)
2	TITLE-ABS-KEY(minor OR underage OR youth OR adolescent OR teenager OR child OR student OR undergraduate OR postgraduate OR young OR youngster)
3	TITLE-ABS-KEY(prevent OR prevention OR intervention OR communication OR campaign)
4	TITLE-ABS-KEY(message OR information)
5	1 AND 2 AND 3 AND 4

4). APA PsycInfo

#	Searches
1	((Title:(electronic cigarette) OR Title:(e-cigarette) OR Title:(vape) OR Title:(vaping) OR Title:(electronic nicotine delivery system)) OR (Subject:(electronic cigarette) OR Subject:(e-cigarette) OR Subject:(vape) OR Subject:(vaping) OR Subject:(electronic nicotine delivery system)) OR (Abstract:(electronic cigarette) OR Abstract:(e-cigarette) OR Abstract:(vape) OR Abstract:(vaping) OR Abstract:(electronic nicotine

	delivery system)))
2	((Subject:(minor) OR Subject:(underage) OR Subject:(youth) OR Subject:(adolescent) OR Subject:(teenager) OR Subject:(child) OR Subject:(student) OR Subject:(undergraduate) OR Subject:(postgraduate) OR Subject:(young) OR Subject:(youngster)) OR (Title:(minor) OR Title:(underage) OR Title:(youth) OR Title:(adolescent) OR Title:(teenager) OR Title:(child) OR Title:(student) OR Title:(undergraduate) OR Title:(postgraduate) OR Title:(young) OR Title:(youngster)) OR (Abstract:(minor) OR Abstract:(underage) OR Abstract:(youth) OR Abstract:(adolescent) OR Abstract:(teenager) OR Abstract:(child) OR Abstract:(student) OR Abstract:(undergraduate) OR Abstract:(postgraduate) OR Abstract:(young) OR Abstract:(youngster)))
3	((Title:(prevent) OR Title:(prevention) OR Title:(intervention) OR Title:(communication) OR Title:(campaign)) OR (Subject:(prevent) OR Subject:(prevention) OR Subject:(intervention) OR Subject:(communication) OR Subject:(campaign)) OR (Abstract:(prevent) OR Abstract:(prevention) OR Abstract:(intervention) OR Abstract:(communication) OR Abstract:(campaign)))
4	((Title:(message) OR Title:(information)) OR (Subject:(message) OR Subject:(information)) OR (Abstract:(message) OR Abstract:(information)))
5	1 AND 2 AND 3 AND 4

5). Web of Science Core Collection including MEDLINE

#	Searches
1	TS=("electronic cigarette" OR e-cigarette OR vape OR vaping OR "electronic nicotine delivery system")
2	TS=(minor OR underage OR youth OR adolescent OR teenager

	OR child OR student OR undergraduate OR postgraduate OR young OR youngster)
3	TS=(prevent OR prevention OR intervention OR communication OR campaign)
4	TS=(message OR information)
5	1 AND 2 AND 3 AND 4
**6). CNKI (China National Knowledge Infrastructure)**	
Search strategy: TKA%='电子烟' AND TKA%=('青少年'+'学生'+'未成年人'+'儿童'+'年轻人'+'青年') AND TKA%=('防控'+'防治'+'传播'+'预防'+'宣传'+'控制'+'干预') AND TKA%=('信息'+'讯息')	

© 2025 Chen Y. et al.
